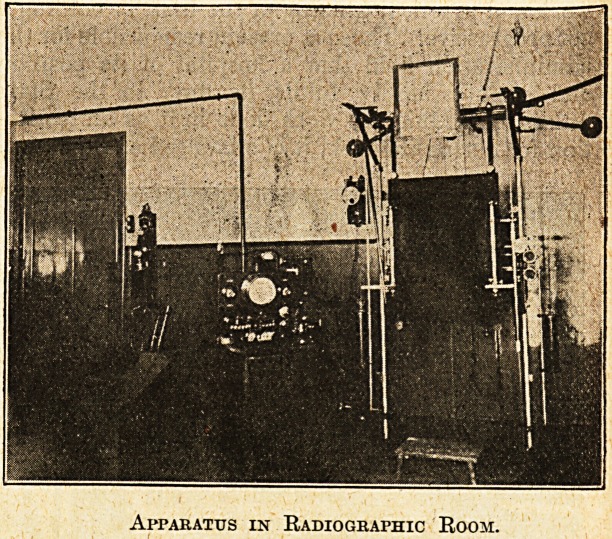# The New Electrical Department at Guy's

**Published:** 1917-02-10

**Authors:** 


### THE NEW ELECTRICAL DEPARTMENT AT GUY'S.

### A Veteran among Hospitals Keeping Up to Date.

GUY'S HOSPITAL, with its medical school for work and practical training of the best, is nothing if not progressive. Those at present responsible for the administration hold that a hospital of its position and repute hardly fulfils its duty to the State unless, in addition to work done in it as an institution for curing the sick, it is organised as a place of

ELECTRICAL DEPARTMENT, GUY'S HOSPITAL.

education, learning, and research. Since 1828, when the medical school was first brought into existence, and especially subsequently to the establishment of a dental school there in 1889, the school has been aided by valuable endowments for medical research, which have added strength and authority to the teaching and practical qualities of Guy's Hospital. The most recent developments include a special department for massage, greatly enlarged and fully equipped, with others for remedial exercises and the therapeutic application of electricity, all dealing with subjects for study and work which the war has brought into prominence. The Guy's authorities have accumulated evidence which demonstrates a considerable demand throughout the country for the services of women trained in the use of *x*-ray apparatus, both as an aid to diagnosis in surgical and medical cases, and as a means of treatment in rodent ulcer, ringworm, and other forms of skin disease. Having regard to the fact that *x*-ray outfits are more widely used in private practice by physicians and surgeons, the call for women able, under their direction, to use such apparatus and to develop and print from skiagraphic negatives, must continuously grow, especially as radiography and dermatology have tested the services technically qualified women are capable of rendering in this connection. Such assistance has proved very valuable in special work of this kind. For these and other reasons the governors at Guy's have decided to utilise educationally the opportunities for training which already exist in the radiographic and actino-therapeutic departments of the hospital, so as to make these branches of practical work accessible to nurses and others capable of being qualified to assist in such work in hospitals, and under practitioners throughout the country. An inspection of the new department enables us to give the following detailed account of what is being done.

The new electrical department, opened in September last to relieve the congestion in the light department and to provide room for new apparatus and facilities for more extended treatment, has by now settled down to a routine of very useful activity. Situated at the top of the medical buildings in a long, airy chamber, which was formerly a dormitory for wardmaids, and on the same floor as the lately completed massage department, it provides ample space for a wide assortment of the most modern apparatus for the electrical treatment of nervous and other conditions. The installation, which cost close upon £200, includes two pantostats, a sinusoid coil, a Schnee bath outfit, radiant-heat baths for shoulders, hands, and knees, and an apparatus for a complete hot-air bath. It is in part divided into cubicles closed by curtains to ensure the necessary privacy.

Since it has been opened there have been 2,629 attendances and 2,750 treatments have been administered. (Some patients receive more than one treatment at a time.) The cases for treatment are chosen from among those attending out-patients and from the wards, selected cases being sent to Dr. Iredell, the head of the department, to indicate appropriate treatment. An interesting branch is the application of Faradaism to cases of traumatic nerve injury in men who have been invalided from the Army for this cause. Already a number have been treated in this way with remarkably successful results, many having almost completely recovered the power of movement in

CLASS ROOM, ELECTRICAL SCHOOL.

the injured limb. Ionisation with various drugs, notably the salicylates, in rheumatoid arthritis, and mercury in obstinate cases of syphilis, is performed on a considerable scale, whilst among other new developments may be mentioned the application of a sinusoidal current to chronic infected sinuses.

The equipment for the latest forms of actinotherapy and *x*-ray treatment at Guy's is now remarkably complete, including as it does a Finsen lamp, four *x*-ray installations, one specially adapted for the treatment of skin diseases, a high-frequency apparatus, static and diathermy machines, etc. In connection with the new department already mentioned and the medical and surgical $x$-ray departments a scheme has been evolved for the training of nurses and others in these branches, which should have quite a special interest at the present time. There is, it is believed, owing to the shortage of men and the great increase in this class of work owing to war conditions, a considerable demand for women who would be competent to take over the electrical department of the smaller hospitals, to run the $x$-ray installation, take skiagraphs, and at the same time administer treatment intelligently under qualified medical supervision. With a view to meeting such a need the authorities of Guy's have therefore availed themselves of the unique teaching facilities offered by the hospital to arrange a course including an adequate amount of theoretical instruction in first principles, and abundant practical tuition in the manipulation and clinical work of each branch. The course is designed to cover six months, and successful students are granted a certificate of efficiency by the hospital. On payment of a slightly higher fee candidates are also prepared for the examination in medical electricity held by the Incorporated Society of Trained Masseuses, though this is only open to those who already hold the Society's massage certificate. The work includes rather more than three months in the radiographic departments, of which about half is devoted to the photographic processes involved—developing, printing, reducing, etc.—and to the necessary clerical work entailed, and the second period to actual clinical demonstrations in the medical and surgical skiagraph rooms by the staff radiographers, Drs. Shenton and Locke.

The remaining time is divided between the actino-therapeutic and electrical departments, a just balance being maintained between theory and practice. Con-

Medical Radiographic Room.

currently with their work in the departments the students attend a course of lectures by Dr. Fison, Lecturer in Physics to the hospital, on electricity and its special application to electrotherapy, and by Dr. Ryffel, Gordon Lecturer in Chemistry to the hospital, on the chemistry of photography and printing. During the whole of their practical work they are under the supervision of the sister of the department, and her assistant, who also holds the Society's certificate.

Altogether the syllabus may be said to be a very full one, and well up to the high teaching standard to be expected at Guy's Last term fourteen pupils attended, and out of that number eleven competed for the Society of Masseuses' certificate and were successful. Though it is early yet to say whether there will be any permanent demand for women so trained,

Apparatus in Radiographic Room.

there can be little doubt that the course will prove of the greatest benefit to those who already hold a massage certificate for the treatment of the particular class of case with which they have to deal.

## Figures and Tables

**Figure f1:**
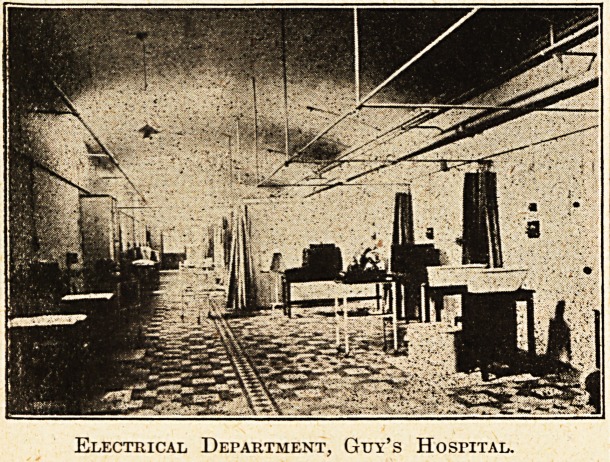


**Figure f2:**
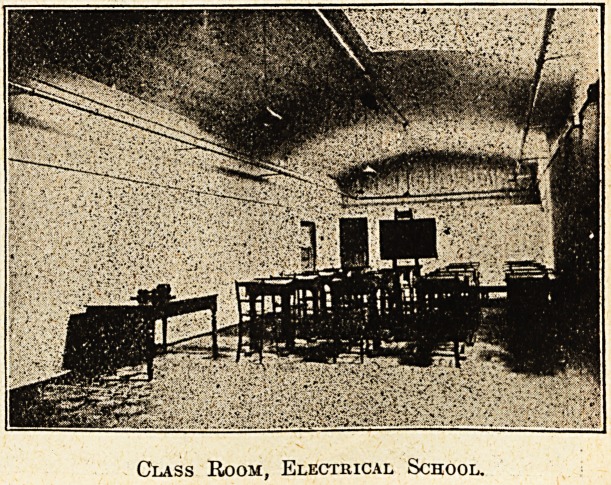


**Figure f3:**
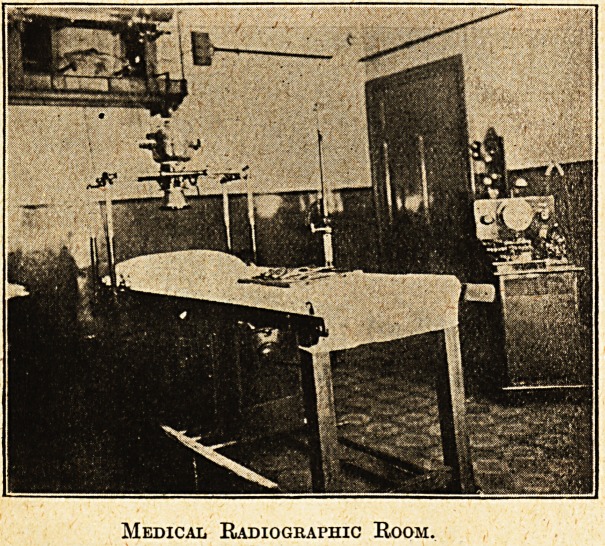


**Figure f4:**